# Simulating latrine conditions to assess perfume performance against malodour

**DOI:** 10.1002/ffj.3450

**Published:** 2018-04-16

**Authors:** Charles Jean‐François Chappuis, Robin Huber, Yvan Niclass, Christian Starkenmann

**Affiliations:** ^1^ Corporate R&D Division Firmenich SA Geneva Switzerland

**Keywords:** climate, model latrine, odour, perfume, toilet malodour

## Abstract

To evaluate perfume performance in toilets, we built model toilets in which critical factors such as background malodour, climate, and airflow were controlled. The models were equipped with an odour generator that injected hydrogen sulfide, methyl mercaptan, butyric acid, para‐cresol, and indole, allowing us to accurately and reliably reconstitute toilet malodour headspace. The malodorant concentrations matched the quantitative headspace analysis performed in African and Indian toilets. The toilet malodour headspace performance was validated by chemical and sensory analysis. Olfactory stimuli were presented to participants in different simulated climates to assess the effect of climate on the perception of odours. The sensory data show that increasing temperature and humidity decreased the intensity ratings of odours without altering their quality. Perfume can be delivered in these toilets by forced evaporation to control the headspace concentration, or by delivery systems such as cellulosic pads, liquids, and powders. Our experimental set‐up allowed us to establish dose–response curves to assess the performance of a perfume in reducing toilet malodour and increasing perceived pleasantness.

## 1 INTRODUCTION

Sanitation is a critical global issue, as 2.5 billion people lack adequate sanitation facilities. To promote the use of toilets and consequently reduce the practice of open defecation that spreads disease, we aim to develop and bring to market affordable perfumes for toilet cleaning and freshening products that have a pleasant smell. This research was linked to the Bill & Melinda Gates Foundation project, ‘Reinvent the Toilet Challenge’.

We initiated our research by identifying molecules that contribute the most to the offending malodour of toilets. Over the course of several field trips we analysed the sludge of toilets in Africa and India, and selected 19 molecules to prepare 40 reconstitutions of toilet malodour.^1^ Using ‘Sniffin’ Sticks’, pen‐like odour dispensers, we validated the most representative reconstitutions in sensory surveys with local people in India and Africa.^2^ We found that a mixture of only four compounds, butyric acid, *p*‐cresol, indole, and dimethyl trisulfide, was necessary to evoke toilet malodour.^2^ We used dimethyl trisulfide because it is a liquid at room temperature with an odour that approaches that of hydrogen sulfide and methyl mercaptan, two important components of toilet malodour.^3^, ^4^ We developed an analytical method to quantify hydrogen sulfide, methyl mercaptan, butyric acid, *p*‐cresol, indole, and skatole in the headspace of latrines in Africa and India.^5^ Knowing the gas‐phase concentrations of the major contributors to toilet malodour allowed us to recreate realistic toilet malodour with synthetic compounds by using olfactometers in the laboratory. Consequently, we were able to test perfumes in a stable and precisely defined background of toilet malodour to find optimal perfume formulations and concentrations.

This approach was not enough, however, to evaluate the performance of perfumes to mask toilet malodour. To develop perfumes that are pleasant in the different climates of Africa and India, we need to understand how temperature and humidity modulates the perception of toilet malodour and perfumes. Temperature affects the evaporation rate of odorant molecules and the resulting gas‐phase concentrations, a key factor that influences the perception of odours. Consequently, the same liquid mixture of odorant compounds may not have the same smell when presented in climates that differ in temperature. Although barometric pressure and humidity have been shown to influence odour detection thresholds,^6^, ^7^, ^8^ little is known about the effect of temperature and humidity on perception itself, regardless of the gas‐phase concentrations of the odorant molecules. More recently, researchers have reported that the integration of temperature signals is partially located in the olfactory systems of rodents and frogs,^9^, ^10^ suggesting that there are interactions between temperature and olfactory systems.

This article describes the development and validation, by chemical and sensory analysis, of an experimental toilet model system that allowed us to evaluate the perception of odours delivered in a controlled manner, in different climates, by varying the temperature and humidity. Inside a climate chamber, we built three model latrines where perfumes can be delivered by delivery systems such as liquids, powders, gels, or cellulosic pads. We also equipped our models with a forced evaporation system inspired by the olfactometer developed by Vuilleumier et al,^11^ allowing us to deliver known gas‐phase concentrations of odorant molecules. The toilet malodour analysed in urine‐diverting toilets (UDTs) in Mukuru (Nairobi) was reconstituted,^5^ and we evaluated the performance of a perfume in covering this malodour in four different climates in sensory panels.

## 2 MATERIALS AND METHODS

### 2.1 Chemicals

The compounds triethylamine, *N*‐ethylmaleimide (NEM), and methyl octanoate were purchased from Sigma‐Aldrich (Buchs, Switzerland), and butyric acid, *p*‐cresol, indole, and l‐cysteine were in‐house products. The solvents propylene glycol, diethyl ether, methanol, ethyl acetate, and acetone were purchased from Carlo Erba (Val de Reuil, France). For methyl mercaptan and hydrogen sulfide, we used nitrogen mixtures at 15 ppm (v/v) in pressurized cylinders purchased from Carbagas (Carouge, Switzerland). Solid‐phase extraction Oasis HLB 1‐g cartridges were purchased from Waters (Montreux‐Chailly, Switzerland). The perfume was an in‐house product with floral tonality, and was a mixture of odorant volatile organic compounds.

### 2.2 Model latrines

**Figure 1:**
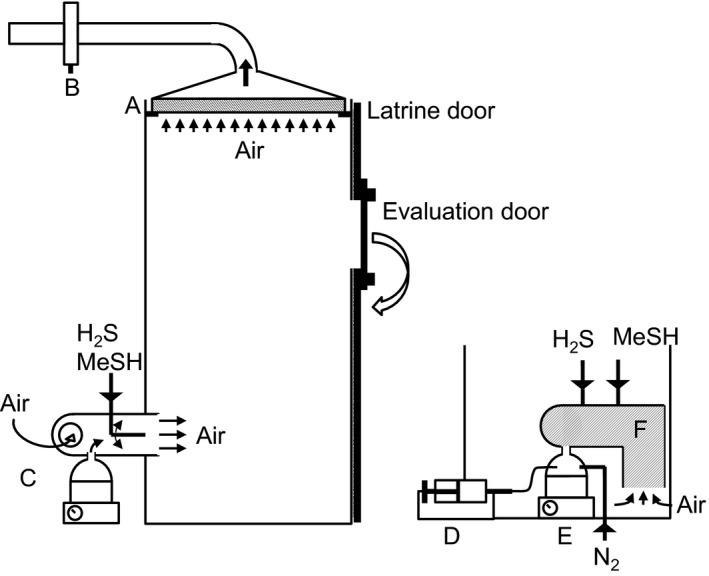
Left, side‐view diagram of a model latrine: A, laminar filter; B, damper; C, odour generator. Right, front view of the odour generator placed behind the model latrine: D, syringe pump; E, round‐bottom glass mounted on the heating plate; F, air inlet pipe guides the air carrying the odour treatments inside the model latrine

Three 1.7‐m^3^ model latrines (1.95 m × 0.985 m × 0.89 m) made of 8‐mm transparent polyethylene terephthalate were placed in a climate chamber, and each latrine was equipped with a 29 cm × 39 cm rotating door to allow the evaluation of odours (Figure 1). The air from the climate chamber entered each latrine via the odour generator placed at a height of 45 cm in the back wall (the odour generator is described in detail below). The air was sucked from the roof of each latrine through a double‐sided 82 cm × 91 cm laminar filter (thick cotton fabric) via a 100‐mm diameter aluminium exhaust tube (Figure 1). The three exhaust tubes were connected to an adjustable fan via a main 100‐mm diameter stainless steel pipe. The airflow of each latrine could be adjusted by changing the suction force produced by the fan if necessary, and could be separately adjusted with a damper (SPI 160; Systemair, Skinnskatteberg, Sweden) placed in the exhaust pipe (Figure 1). A hot‐wire anemometer was placed in the main exhaust pipe to measure the main exhaust flow. The airflow inside this pipe was maintained at 51 m^3^/h, distributing 17 m^3^/h in each latrine. The airflow and resulting air changes per hour (roughly 10) were in the range of measurements made in a ventilated improved pit latrine.^12^

### 2.3 Odour generator

To force the evaporation of liquids we modified the lower chamber of the olfactometer, as described by Chappuis et al.^5^ A 150‐L/h nitrogen flow flushed a 500‐ml round‐bottom flask, where liquids were introduced via a polytetrafluorethylene (PTFE) capillary linked to a 1‐ml polypropylene syringe (Figure 1). The flow rate of liquids was delivered by a syringe pump and could range from 0.04 to 10 mL/h. The flask was covered with a glass hat and heated to 160 °C with a stainless steel heat‐on block mounted on a 600W heating plate (Figure 1). The outlet of the flask (2‐mm inner diamater, i.d.) was placed in a stainless steel pipe (15‐cm long, 109‐mm diameter) that crossed the back wall of the latrine. This pipe was connected to an aluminium exhaust pipe (80‐cm long, 127‐mm diameter) placed in the back wall (Figure 1) to avoid sucking heated air inside the latrine. The nitrogen that was enriched with odorant molecules mixed with the air that entered the latrine. In addition to the forced evaporation release systems, two stainless steel tubes were soldered inside the pipe to hold 6‐mm PTFE tubes to release methanethiol and hydrogen sulfide from pressurized cylinders. The PTFE tubes were closed with Parafilm and two 0.8‐mm openings per tube were pierced with a needle (Figure 1).

The nitrogen flow rate of each latrine was controlled with three rotameters (25–250 L/h; Krohne, Duisburg, Germany). The flow rates of methanethiol and hydrogen sulfide were controlled independently with six mass flow meters (three toilets, two gases) (Red‐y; Vögtlin Instruments AG, Aesch, Switzerland).

### 2.4 Climate chamber

The climate chamber dimensions were 3.42 m × 2.95 m × 2.5 m, resulting in a volume of 25 m^3^. The temperature and humidity of the climate chamber was controlled in a closed cycle of 540 m^3^/h. Fresh air entered the chamber at a rate of 51 m^3^/h, and air left the chamber at the same rate. The working range for temperature and relative humidity (RH) was 12–45 °C and 30–90% RH, respectively. The climate chamber was equipped with temperature and RH probes placed at the entrance and outlet of the temperature and humidity controlling cycle. The data from the probes at the entrance were recorded every 5 min, allowing the measurement of temperature and RH of the air inside the climate chamber during the experiments. Moreover, we placed a probe (Traceable^®^ hygrometer; VWR International, Radnor, PA, USA) inside the latrine to punctually measure the RH and temperature to ensure that the differences in temperature and RH between the air inside the climate chamber and the air inside the latrines were minimal. We aimed to maintain the temperature difference below 1.5 °C and the RH difference below 5%.

### 2.5 Participants

The participants for the sensory panels were employees from the research centre at Firmenich SA (Geneva, Switzerland). Ten sessions were organized and the number of participants for each session was as follows: 26, 24, 26, 27, 26, 30, 25, 27, 25, and 23. The participants signed a consent form before participating in the study. The consent form and experimental protocol were approved by the internal review board of Firmenich in agreement with the Declaration of Helsinki for medical research involving human subjects. All signed forms were kept by Dr Charles Jean‐François Chappuis.

### 2.6 Stimuli

The participants were exposed to six odorant mixtures delivered in latrines in four different climates. The odorant mixtures were Mukuru (Nairobi) UDT malodour alone, the perfume (Floral tonality, in‐house) alone, and mixtures of the malodour and the perfume released at four different gas‐phase concentrations (0.18, 0.54, 1.62, and 4.9 μg/L).

The malodour was reconstituted from Mukuru toilets because they contain all of the significant molecules and come from well‐maintained toilets. It was composed of hydrogen sulfide, methanethiol, butyric acid, *p*‐cresol, and indole, the gas‐phase concentrations of which were 0.26, 0.018, 0.004, 0.0027, and 0.00018 μg/L, respectively. Hydrogen sulfide and methanethiol were released from pressurized cylinders at 20.8 and 9.8 L/h, respectively. The remaining malodour products were released in the latrines by forcing the evaporation of a propylene glycol solution that contained 0.775, 0.526, and 0.035 mg/mL of butyric acid, *p*‐cresol, and indole, respectively. The perfume was released in pure form in the forced evaporation chamber, resulting in a gas‐phase concentration of 4.9 μg/L. The lower gas‐phase concentration of the perfume was achieved by diluting it in propylene glycol. The gas‐phase concentrations of 0.18, 0.54, and 1.62 μg/L were obtained with 3.62, 11.13, and 33.31% (w/w) propylene glycol solutions, respectively. To release the malodour and the perfume in the same latrine, we connected two syringes via two PTFE capillaries to the same forced evaporation chamber. One contained the malodour solution and the other contained the perfume, either in pure form or diluted in propylene glycol. Both syringes were mounted on the same syringe pump and their pistons were pushed at 0.088 mm/h, resulting in a release rate of 0.088 mL/h. When the malodour or the perfume was presented alone, pure propylene glycol was injected into the forced evaporation chamber with the second syringe. Each odour was presented in four climates: 22 °C at 30% RH, 22 °C at 80% RH, 35 °C at 30% RH, and 35 °C at 80% RH.

### 2.7 Sensory protocol

The participants were randomly exposed to the odour stimuli in the different climates. As only three latrines were available, the six odours were split into two groups, each containing the malodour alone or the perfume alone, the malodour plus a low dose of perfume, and the malodour plus a high dose of perfume. The first and the last sessions were used as controls to assess the reliability of the panel, and were composed of the malodour alone, the perfume alone, and a mixture of both. The participants entered the climate chamber and directly evaluated the odour of the three latrines by answering a paper questionnaire made with the software fizz (Biosystems, Courtenon, France). The questionnaire is available in the Figures S1 and S2. They re‐evaluated the odour of each latrine after a 3‐min adaptation to the climate. They were asked to rate, on linear scales of 0–10, the pleasantness from ‘I don't like’ to ‘I like’, the familiarity from ‘not familiar’ to ‘very familiar’, the intensity from ‘no odour’ to ‘very strong’, the faecal/toilet character from ‘not faecal/toilet’ to ‘very faecal/toilet’, and whether they wanted to enter the latrine from ‘not at all’ to ‘very willingly’.

### 2.8 Headspace analysis

The Mukuru malodour was released in the model latrines as described above. The climate was set to 25 °C at 50% RH. The compounds released into the air were collected with Oasis cartridges conditioned with 20 mL of deionized water, 20 mL of methanol, 20 mL of acetone, and 20 mL of diethyl ether, and dried in an oven at 50 °C for 1 h. Hydrogen sulfide and methyl mercaptan were derivatized with NEM in Oasis cartridges loaded with 2 mL of diethyl ether containing 25 mg of NEM and 100 μL of triethylamine, and dried at 50 °C for 1 h. The air was pumped at 1 L/min through the cartridges by using GilAir Plus pumps connected with silicon tubes. The volume of the samples was 100 L. Three cartridges were used to sample the air of one latrine: one cartridge was placed in the centre of the model latrines, the second was placed 23 cm from the evaluation door, and the third was placed deep at the top right of the latrine (171 cm from the ground). The cartridges were desorbed with 10 mL of diethyl ether added to 100 μL of 10 ng/μL methyl octanoate (internal standard, IS) solution in ethyl acetate. To remove the excess NEM, we washed the eluate with 3 mL of 10 mg/mL of l‐cysteine solution in water buffered at pH 8 with 0.1 M potassium phosphate. The water phase was removed and the organic phase dried with sodium sulfate. The water phase was acidified with 100 μL of a 37% HCl solution in water, and butyric acid was extracted with 4 mL of diethyl ether added to 100 μL of IS. Prior to injection in the GC‐MS, both organic phases were gently concentrated to 1 mL under argon flow. The analysis was performed by injecting 1 μL of the eluate into the GC‐MS as described below.

### 2.9 Headspace analysis calibration using an olfactometer

Using an olfactometer, we created headspaces with known concentrations of butyric acid, indole, *p*‐cresol, methyl mercaptan, and hydrogen sulfide to calibrate our analytical method, as described by Chappuis et al.^5^ Briefly, air with known quantities of compounds was sampled with Oasis cartridges loaded with the derivatization agent NEM (described above) at the outlet of the olfactometer. Methyl mercaptan and hydrogen sulfide were released into the olfactometer from pressurized cylinders containing a mixture of 15 ppm of both sulphur compounds in nitrogen. The flow of both sulphur compounds was controlled with rotameters (Vögtlin TV 100). Butyric acid, indole, and *p*‐cresol were released by forcing the evaporation of propylene glycol solutions from a 1‐ml polypropylene syringe mounted on a syringe pump delivering 0.101 mL/h. The solution was introduced into the lower chamber, which was heated to 150 °C by using an oil bath. Nitrogen was introduced into the lower chamber at 60 L/h to collect the products that evaporated, and was mixed with the airflow of the upper chamber. The airflow was set at 540 L/h and humidified by bubbling in a water‐jacketed wash bottle filled with distilled water. The upper chamber of the olfactometers was water‐jacketed and its temperature was maintained at 29 °C with a water bath. At the outlet of the olfactometers, the temperature was 30 °C and the RH was 40%. The resulting concentrations of methyl mercaptan and hydrogen sulfide compounds in the olfactometer were 0.1, 0.05, 0.0250, and 0.0125 μg/L. The resulting concentrations of butyric acid, *p*‐cresol, and indole were 0.0001, 0.001, 0.01, and 0.1 μg/L.

### 2.10 Gas chromatography−mass spectrometry (GC‐MS)

A GC 6890 N (Agilent, Palo Alto, CA, USA) was used to identify the compounds. A fused silica SPB‐1 capillary column (30 m × 0.25 mm i.d., 0.25‐μm film thickness; Supelco, Bellefonte, PA, USA) was mounted in the GC. The carrier gas was He (52 kPa) and the injector temperature was set at 250 °C. Injections were made with a Combi‐Pal autosampler (Zwingen, Switzerland). To analyse butyric acid, *p*‐cresol, indole, and NEM derivatives of methyl mercaptan and hydrogen sulfide, we held the initial oven temperature at 50 °C for 5 min and then increased it at 5 °C/min to 250 °C, in split mode 1/5. The GC was coupled to an MS 5975B Inert XL MSP from Agilent. The mass spectra in electron impact mode were measured at 70 eV in selected ion monitoring (SIM) mode. The ions that were monitored were butyric acid (60), *p*‐cresol (107), indole (117), NEM‐*S*‐CH_3_ (127), and NEM‐*S*‐NEM (127).

### 2.11 Data analysis

The questionnaires were scanned and the data stored in fizz and analysed with r (https://cran.r-project.org). The response variables – pleasantness, enter the latrine, intensity, familiarity, and faecal character – were analysed by using analysis of variance (ANOVA), and any significant effect was confirmed with the non‐parametric Kruskal–Wallis test. Pairwise comparison tests were made with the Tukey honest difference test (tukey hsd function in r). The relationship between the pleasantness of the different odour treatments and willingness to enter the latrines was investigated with linear models. Pleasantness ratings from the odour treatments and pleasantness ratings from the climates were analysed with linear models. The level of significance was set at *P* < 0.05. To determine the concentrations of malodour compounds in the gas phase of the model latrines, we established calibration curves by using linear models on the ratio of the peak area of volatiles and IS as a function of the gas‐phase concentrations in the olfactometer. Using these calibration curves and the inverse prediction function in r (chemcal package), we were able to predict the gas‐phase concentrations inside the model latrines.

## 3 RESULTS

**Figure 2:**
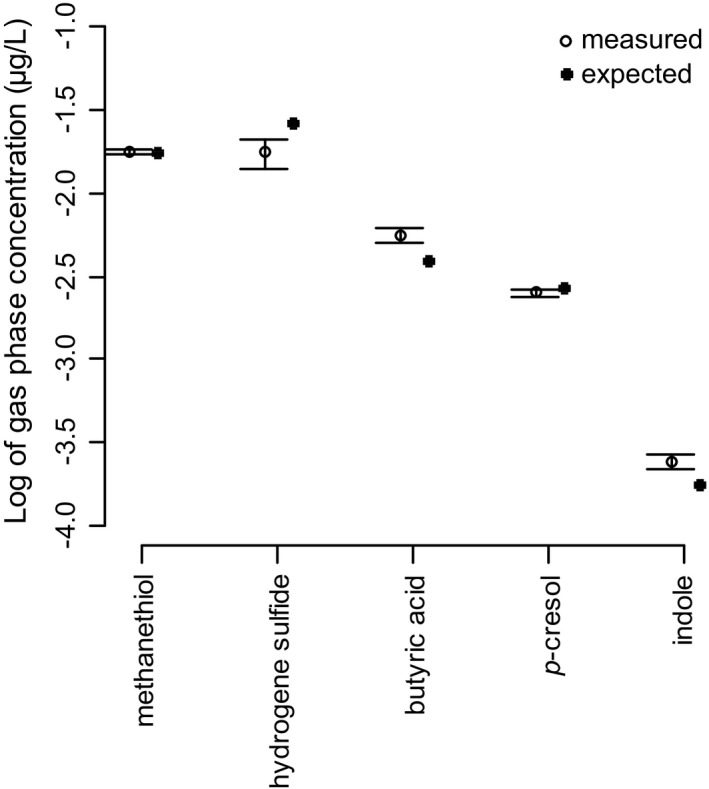
Odorant homogeneity within a toilet and between three toilets. Mean ± standard deviation (SD) of measured gas‐phase concentrations compared with the expected values (*n* = 9)

We created the typical Mukuru faecal toilet malodour through a controlled release of methanethiol, hydrogen sulfide, butyric acid, *p*‐cresol, and indole by spraying the gas and vaporizing the liquids in a hot chamber flushed with nitrogen (Figure 1).^5^ The headspace was analysed in three different locations in each of the three toilets. The results of the quantifications, compared with the expected concentrations, are shown in Figure 2. The target concentrations of methanethiol, hydrogen sulfide, butyric acid, *p*‐cresol, and indole were attained at 101, 66, 130, 93, and 138%, respectively, of the expected values, which we obtained by knowing how much of each compound had been injected. The standard deviations showed a relatively small interval, indicating a homogeneous headspace inside the toilets in addition to a reproducible headspace between the toilets.

When the subjects stepped into the climate chamber, they were exposed to the temperature and humidity set up for the experiment; therefore, for the present study, they were asked to make a first evaluation of the odour directly after entering the chamber, and to make a second evaluation after a few minutes of adaptation to the climate. Adaptation had no significant effect on the criteria used to evaluate the odour. The data with and without adaptation were then averaged.

**Figure 3:**
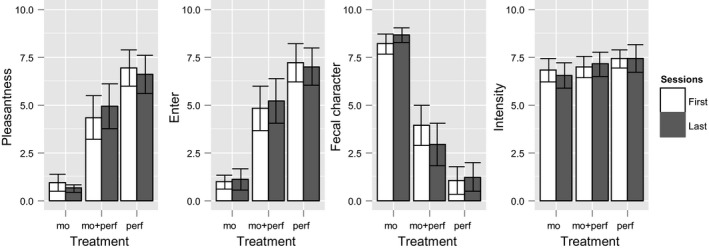
Validation of the sensory protocol. Mean ± 95% confidence interval (95% CI) of pleasantness, willingness to enter, faecal character, and intensity of odour ratings. Abbreviations: mo, malodour; perf, 4.9 μg/L perfume. The climate was 22 °C and 30% relative humidity

Four descriptors were proposed to the subjects: pleasantness, willingness to enter the toilet, faecal character, and intensity. The panel was reliable, as the results obtained with the panel when we repeated the first and last sessions did not significantly differ (Figure 3). Considering that the panel is reliable and the olfactory stimuli were not delivered in the same order during the first and the last session, we did not find any bias resulting from a potential intrinsic odour of a particular model latrine. No odour was detected when syringe pumps stopped or when the gas was switched off. Therefore, to save panelists time, sniffing blanks were not performed.

**Figure 4:**
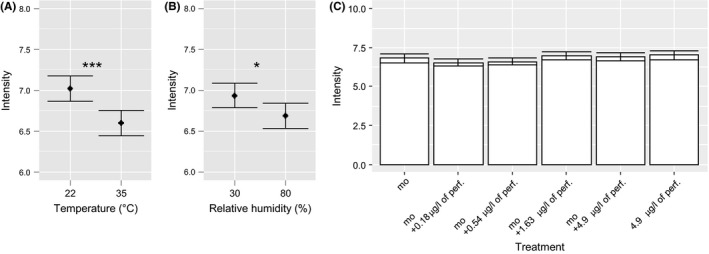
A, Mean ± 95% confidence interval (95% CI) of intensity evaluation at 22 and 35 °C; the data obtained with all odours at 30 and 80% humidity are combined. B, Intensity evaluation obtained with all odours at 30 and 80% humidity; the data at 22 and 35 °C are combined. C, Evaluation of the intensity of the sensory stimuli with a constant Mukuru urine‐diverting toilet (UDT) malodour and increasing perfume concentration (all climates combined). Asterisks show the levels of significant differences in the means: ***P < 0.0001, *P < 0.05

The climate significantly affected the intensity, but had no significant effect on the other criteria of pleasantness, familiarity, faecal character, and willingness to enter the latrines. According to the statistical analysis, the increase in temperature significantly decreased the overall intensity (ANOVA, *P* < 0.0001; Kruskal–Wallis, *P* < 0.001; Figure 4A). Similarly, but to a lesser extent, an increase in humidity significantly decreased the intensity (ANOVA, *P* < 0.05; Kruskal–Wallis, *P* < 0.05), as shown in Figure 4B. A combined increase in humidity and temperature did not significantly further depress the overall intensity, however. The significant effect of temperature was mainly linked to differences in means obtained with the malodour alone, with the mixtures of the malodour and the highest perfume concentration, and with the perfume alone. The significant effect of humidity was mainly due to the mixtures of the malodour and the highest perfume concentration, and to the perfume alone. The intensity for malodour alone, perfume alone, and the mixtures of malodour in addition to increasing perfume concentrations were similar, as pairwise comparisons (Tukey honest difference) revealed no significant differences (Figure 4C).

**Figure 5:**
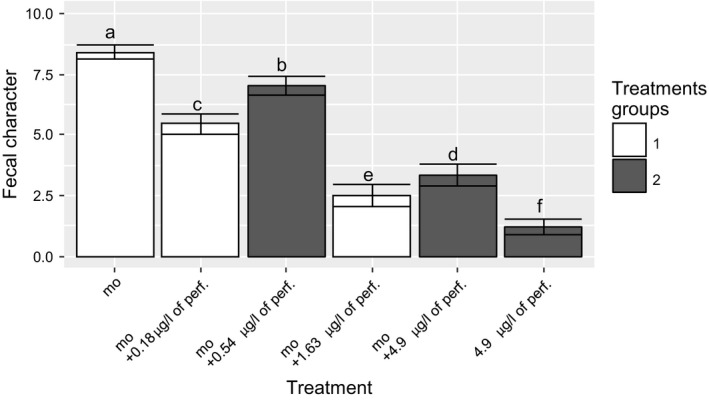
Sensory evaluation of the impact of the reference malodour or perfume on the response variable of faecal character. Mean ± 95% confidence interval (95% CI) of the faecal character of the odour treatments. White and dark‐grey bars indicate groups of olfactory stimuli. Means with different letters are significantly different following a pairwise test based on analysis of variance (ANOVA). Abbreviations: mo, malodour; perf, perfume

**Figure 6:**
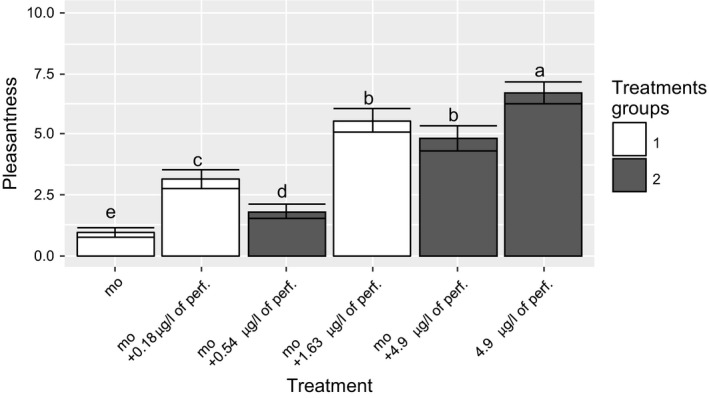
Sensory evaluation of the impact of the reference malodour or perfume on the response variable of pleasantness. Mean ± 95% confidence interval (95% CI) of the pleasantness ratings as a function of the odour treatments. White and dark‐grey bars indicate groups of olfactory stimuli. Means with different letters are significantly different following a pairwise test based on analysis of variance (ANOVA). Abbreviations: mo, malodour; perf, perfume

The faecal character was significantly depressed when the perfume concentration was increased, as shown in Figure 5. As opposed to the direction of the faecal character ratings, the pleasantness ratings increased significantly as a function of increasing concentrations of perfume (Figure 6). Because we made three model toilets and tested six olfactory stimuli, we split the six stimuli into two groups: groups 1 and 2 corresponding to white and grey bars in Figures 5 and 6. A direct comparison between both groups is not possible, as explained in the discussion, so only the trend can be considered.

**Figure 7:**
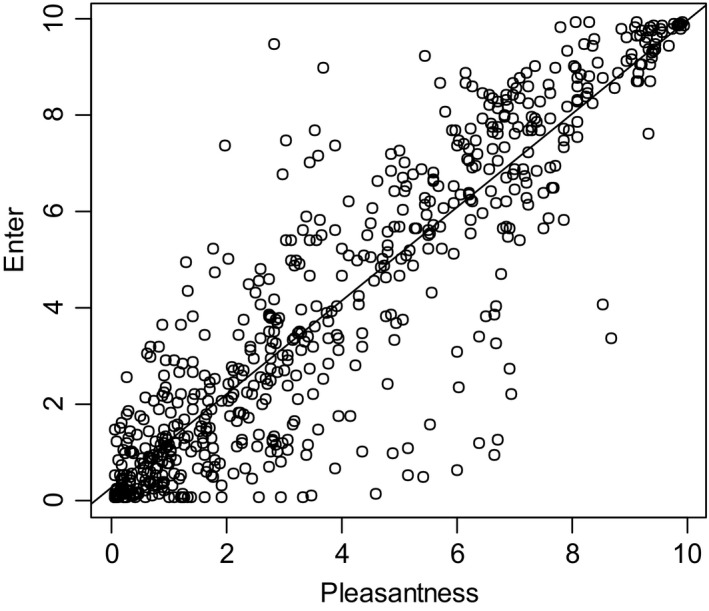
The willingness‐to‐enter ratings as a function of the pleasantness ratings. The line shows the linear model that predicts the willingness‐to‐enter ratings by the pleasantness ratings

A similar result was obtained with the ratings of willingness to enter the latrines compared with the pleasantness ratings. Ratings of willingness increased significantly as a function of increasing perfume concentrations. The willingness to enter the latrine was strongly correlated with pleasantness (Figure 7, linear model, slope = 0.97, *P* < 0.0001; intercept = 0.27, *P* < 0.001, adjusted *R* ^2^ = 0.7986). This linear model explained roughly 80% of the variance of the willingness‐to‐enter ratings with the pleasantness ratings on the abscissa. The effect of the treatment groups on the willingness‐to‐enter ratings was much lower than that on the pleasantness ratings.

The climate chamber was set for four climate conditions: 22 °C at 30% RH, 22 °C at 80% RH, 35 °C at 30% RH, and 35 °C at 80% RH. The temperature and RH conditions were reached by using the temperature controlling system (Table 1). The temperatures and the RHs inside the climate chamber and inside the model latrines were less than 1.5 °C and 5%, respectively (Table 1).

**Table 1:** Measurements of temperature and relative humidity (RH) inside the climate chamber and inside the latrine

Temperature set (°C)	RH set (%)	Average temperature of climate chamber (°C)	Standard deviation of temperature of climate chamber (°C)	Average RH of climate chamber (%)	Standard deviation of RH of climate chamber (%)	Temperature of latrine (°C)	RH of latrine (%)
22	30	22.0	0.6	32.6	3.1	23.0	31.3
35	30	35.0	0.2	30.4	0.9	34.4	28
22	80	22.0	0.2	73.0	2.5	22.8	75.5
35	80	35.0	0.2	76.0	2.2	34.8	81

## 4 DISCUSSION

We were able to validate our gas‐phase reconstitution of the Mukuru toilet malodour with our analytical method, and we demonstrated that the headspace was reproducible and homogenous. Moreover, we demonstrated that the changes in the perception of odours presented at the same concentrations in different climates are limited to variations in the intensity ratings. The intensity of odour treatments decreased when the temperature and the humidity were increased. The other criteria, pleasantness, familiarity, faecal character, and willingness to enter the latrines, were not affected by the climate modifications. Our results also showed that our experimental set‐up allowed the construction of dose–response curves, although the results were strongly affected by grouping olfactory stimuli.

To our knowledge, this study is the first report on the perception of odours presented at the same concentrations in different climates that varied in both temperature and humidity. Temperature clearly affected the evaporation rates of odorant compounds, and then changed the perception of odours. Our experimental set‐up delivered the odours with a forced evaporation system, freeing us from the effect of temperature on the evaporation rates of odorant compounds and allowing us to focus on perception. Our results showed that temperature and humidity increments depress the perceived intensity of the odour treatments. Previous research showed that atmospheric pressure and humidity affected the detection threshold of pure compounds released by ‘Sniffin’ Sticks’. Kuehn et al showed that in hypobaric conditions, the olfactory detection threshold increased compared with that in normal baric conditions.^6^ The authors thought that the decrease in olfactory sensitivity could be explained by the hypobaric condition, which implies gas expansion that consequently reduces odorant concentration. In contrast, they found that humidity increased olfactory sensitivity by decreasing the olfactory detection threshold of single compounds. We showed that humidity slightly decreased the perceived intensity of odours, suggesting a decrease in olfactory sensitivity. However, we tested mixtures of compounds released by a forced evaporation system, making it difficult to compare results between the two studies. We demonstrated for the first time that temperature affects the perception of odours by decreasing their intensity. A first explanation could be the effect of the vapour pressures of the odorant compounds, which increased with temperature. Inside the nose, some of the odorant molecules leave the air and cross the mucus above the olfactory epithelium to reach the receptors of the olfactory neurons.^13^ When temperature increases, the vapour pressure also increases, and this reduces the number of molecules that cross the mucus, resulting in a decrease in intensity; however, mammals maintain a steady body temperature and the nose plays a crucial role as a powerful air‐conditioning machine to keep near‐alveolar conditions at the outlet of the nose on inhalation.^14^, ^15^ For example, Farley and Patel demonstrated that breathing air at –17 °C resulted in a temperature of 32.7 °C in the pharynx.^16^ Consequently, the difference in the climates in our experiments are not likely to result in a change in temperature inside the nose that caused a significant change in the vapour pressure of the compounds tested. Another explanation for the decrease in intensity ratings as a result of the effect of temperature on odour perception could be that olfactory and heat signals share, in part, a common neuronal network.^9^, ^10^ In our study, the effect of temperature and humidity on perception was limited to variable intensity only. This result lessens the importance of the effect of both physical variables on the perception of odours in our study. The evaporation rate is much more sensitive to changes in temperature, resulting in different gas‐phase concentrations of odorant compounds, and thus modifying perception. These model latrines were also designed so that we could evaluate the delivery systems at the perception and analytical levels to ensure a sufficient gas‐phase concentration of perfumes to reduce the malodour of toilets.

Our gas‐phase reconstitution of Mukuru UDT malodour was evaluated as strong and very faecal. This is in line with our previous work and with research showing the importance of butyric acid, *p*‐cresol, indole, hydrogen sulfide, and methyl mercaptan as components of human faecal odour.^2^, ^3^, ^17^ Releasing the sulphur compounds stably is challenging because they are unstable gases. We overcame this problem by releasing mixtures of both compounds in nitrogen from pressurized cylinders. In addition to these channels for releasing gases, we equipped our model latrines with a forced evaporation system to deliver compounds that are liquid or solid at room temperature. Moreover, the odours were delivered in a continuous flow, ensuring the stable concentration of odours over time. This method greatly facilitated the sensory panels because the first panelist smelled a mixture that was equal to that smelled by the last panelist. It also allowed us to reconstitute a realistic olfactory background of toilets, and to stably deliver known quantities of a perfume to evaluate its performance to reduce the malodour. We could show that the total concentration of this particular perfume should be above 1.6 μg/L to efficiently reduce our strong background malodour, and to obtain positive pleasantness ratings; however, we showed that splitting the olfactory stimuli into two treatment groups created a bias in the data. In fact, the perfume performed less well in group 2 than in group 1, although the gas‐phase concentrations of perfume were higher in group 2 than in group 1. Both groups did not have the same olfactory stimuli. Group 1 consisted in the malodour alone, and to two mixtures of the malodour and the perfume. Group 2 consisted in to the perfume alone, and to two mixtures of the malodour and the perfume. Participants may have compared the odour of the three latrines and adjusted their ratings after the evaluation of the three latrines, resulting in lower ratings in group 1 and higher ratings in group 2, when the malodour alone was present. The malodour alone provoked a negative experience in participants, as shown by our data. This response could reduce the time spent analysing and the attention needed for a proper analysis of the malodour, as proposed by Herz,^18^ and also by Ferdenzi et al.^19^ We found that pleasantness ratings were positively correlated with the concentration of perfume in the presence of the malodour background. This could be explained by the fact that the faecal character was negatively correlated with the concentration of the perfume, and that the perfume alone was pleasant. Toilet malodour is generated by decomposing material, and is generally perceived as disgusting.^20^, ^21^, ^22^ The odours of faeces, vomit, or decaying material are associated with potential microbial threats, and they elicit avoidance.^23^ Thus, it was not surprising that the willingness to enter the model latrine was positively correlated with pleasantness. This result suggests that a pleasant odour should promote the use of clean and well‐maintained toilets as, among other factors, odour plays an important role in the perception of the cleanliness of toilets.^24^

We improved the analytical method described by Chappuis et al^5^ to quantify hydrogen sulfide, methyl mercaptan, butyric acid, *p*‐cresol, and indole in the gas phase. In the previous method, the air sample was pumped through a wash bottle filled with buffered water containing the derivatization agent NEM. The water trapped the target molecules, and sulphur compounds were derivatized with NEM. The water was then loaded on Oasis HLB cartridges. In our study, instead of using water as a trap, we directly sampled the air through the Oasis cartridges previously loaded with NEM and triethylamine. To our knowledge, this is the first time that solid‐phase extraction cartridges such as Oasis HLB have been used to directly collect volatile organic compounds and sulphur compounds from the air. These cartridges are mainly used to extract compounds from liquid.^25^, ^26^, ^27^, ^28^, ^29^ They contain a solid phase made of a co‐polymer of *N*‐vinylpyrrolidone and divinylbenzene, giving them interesting hydrophilic and lipophilic properties. The phase is wettable because of the *N*‐vinylpyrrolidone, and divinylbenzene provides the reversed‐phase retention of analytes.^30^ Here, we showed that air samples can be pumped through Oasis HLB cartridges at a rate of 1 L/min, and that the sorbent was suitable to trap butyric acid, *p*‐cresol, and indole, as well as being suitable to derivatize sulphur compounds with NEM and trimethylamine. This new method has the advantage of reducing the quantity of laboratory materials needed in the field, and was sensitive enough to quantify the target molecules at concentrations found in our model latrines. Using this analytical method, we demonstrated that the odour generators released the quantity of volatiles that we targeted with relatively high accuracy. In addition to filters distributing air velocities across the model latrines, the level of turbulence inside the odour generators and inside the model latrines was high enough to obtain homogenous gas‐phase concentrations of volatile organic compounds. This level of homogeneity is not expected in the field, but it ensures reliable and stable results from laboratory experiments, which are crucial steps before conducting field experiments.

By controlling critical factors such as the background malodour, climate, and airflow, we developed a unique tool that accurately reproduces the toilet environment. This allows us to evaluate the performance of perfumes and various release systems (cellulosic pads, detergents, etc.) in stable and realistic conditions at the analytical and sensory level, helping us to develop products that are efficient in the toilet environment. The environment that we can reproduce, however, is not limited to that of toilets. The climate chamber and the odour generator, when equipped with a forced evaporation and gas release system, can produce a great variety of background odours and climates.
