# Supplementary material for: Simulating latrine conditions to assess perfume performance against malodour
Source: Flavour Fragr J. 2018 Apr 16;33(4):313–21. doi: 10.1002/ffj.3450 (PMC6049876; doi:10.1002/ffj.3450)
Supplement: Supplementary file 2 [file FFJ-33-313-s002.docx]

Supplementary material 2
